# Utilizing virtual experiments to increase understanding of discrepancies involving in vitro-to-in vivo predictions of hepatic clearance

**DOI:** 10.1371/journal.pone.0269775

**Published:** 2022-07-22

**Authors:** Preethi Krishnan, Andrew K. Smith, Glen E. P. Ropella, Lopamudra Dutta, Ryan C. Kennedy, C. Anthony Hunt

**Affiliations:** 1 Bioengineering and Therapeutic Sciences, University of California, San Francisco, San Francisco, CA, United States of America; 2 Tempus Dictum, Inc., Olympia, WA, United States of America; Instituto Politecnico Nacional, MEXICO

## Abstract

Predictions of xenobiotic hepatic clearance in humans using in vitro-to-in vivo extrapolation methods are frequently inaccurate and problematic. Multiple strategies are being pursued to disentangle responsible mechanisms. The objective of this work is to evaluate the feasibility of using insights gained from independent virtual experiments on two model systems to begin unraveling responsible mechanisms. The virtual culture is a software analog of hepatocytes in vitro, and the virtual human maps to hepatocytes within a liver within an idealized model human. Mobile objects (virtual compounds) map to amounts of xenobiotics. Earlier versions of the two systems achieved quantitative validation targets for intrinsic clearance (virtual culture) and hepatic clearance (virtual human). The major difference between the two systems is the spatial organization of the virtual hepatocytes. For each pair of experiments (virtual culture, virtual human), hepatocytes are configured the same. Probabilistic rules govern virtual compound movements and interactions with other objects. We focus on highly permeable virtual compounds and fix their extracellular unbound fraction at one of seven values (0.05–1.0). Hepatocytes contain objects that can bind and remove compounds, analogous to metabolism. We require that, for a subset of compound properties, per-hepatocyte compound exposure and removal rates during culture experiments directly predict corresponding measures made during virtual human experiments. That requirement serves as a cross-system validation target; we identify compound properties that enable achieving it. We then change compound properties, *ceteris paribus*, and provide model mechanism-based explanations for when and why measures made during culture experiments under- (or over-) predict corresponding measures made during virtual human experiments. The results show that, from the perspective of compound removal, the organization of hepatocytes within virtual livers is more efficient than within cultures, and the greater the efficiency difference, the larger the underprediction. That relationship is noteworthy because most in vitro-to-in vivo extrapolation methods abstract away the structural organization of hepatocytes within a liver. More work is needed on multiple fronts, including the study of an expanded variety of virtual compound properties. Nevertheless, the results support the feasibility of the approach and plan.

## Introduction

In vitro-to-in vivo extrapolation (IVIVE) methods, employing hepatocytes or liver microsomes, are widely used in toxicology and during preclinical drug development to predict the hepatic clearance of xenobiotics, particularly in humans. Despite more than a decade of research, reliably accurate predictions are not yet achievable. Underprediction of hepatic clearance is the most daunting problem [1]. Discussions highlight the importance of identifying responsible mechanisms and using that knowledge to develop improved IVIVE methods [1–3]. However, the realities and uncertainties of working with isolated hepatocytes and scaling-derived measures to humans present numerous impediments to disentangling those mechanisms [3, 4].

**Fig 1:**
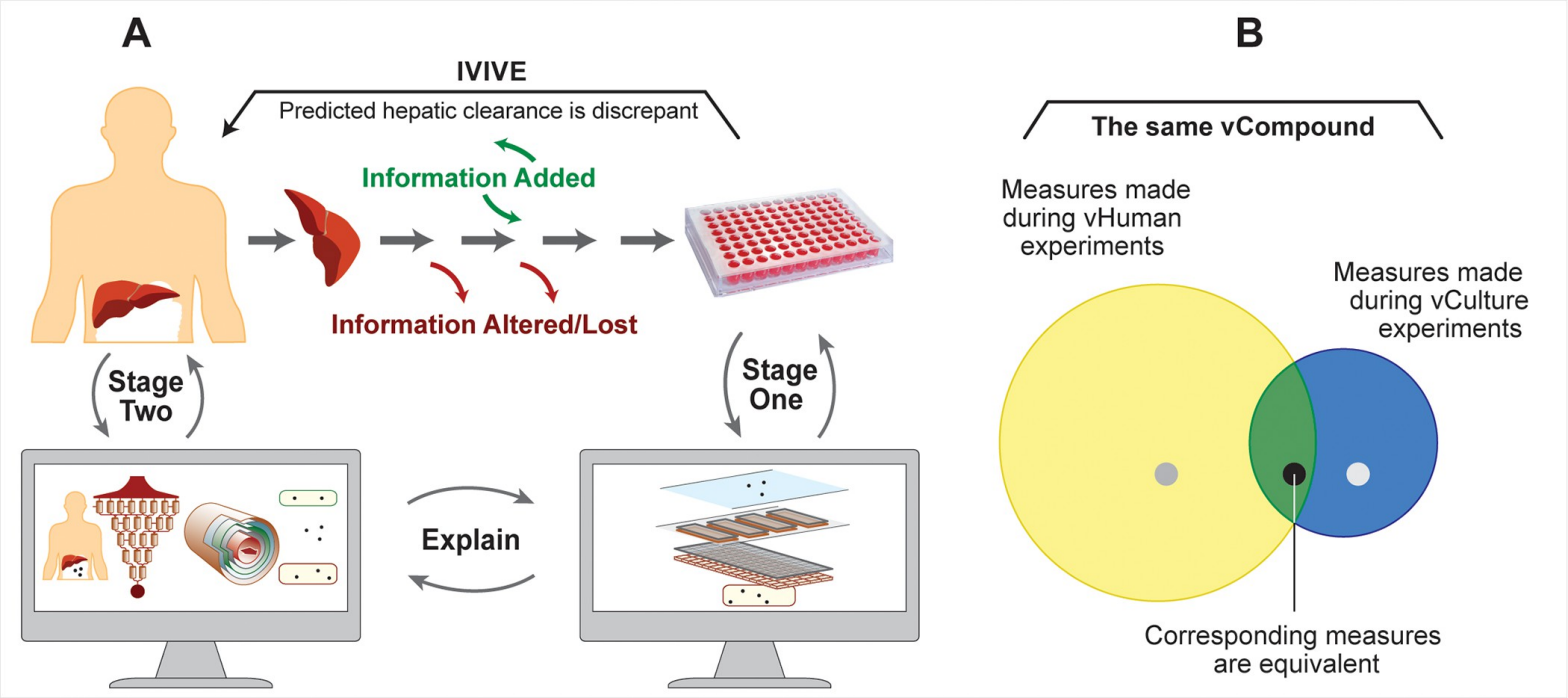
Discovering plausible mechanism-based explanations contributing to IVIVE discrepancies. (A) Starting with a failed IVIVE prediction of hepatic clearance, the plan requires vCulture and vHuman analogs that have achieved multiple validation targets. Stage One establishes a direct quantitative mapping between measures of removal of vCompound during vCulture experiments and the data used to compute intrinsic clearance. Independently, Stage Two establishes a direct quantitative mapping between vCompound removal measures during vHuman experiments and the data used to compute hepatic clearance. We then explain vCompound disposition and removal differences and use that information to posit explanations for the observed IVIVE discrepancy. (B) The blue and yellow circles illustrate comparable per vHPC measures of disposition and removal for different vCompound properties. Measures from each system are quantitatively equivalent within the area of overlap. The black circle illustrates a vCompound that achieves the cross-system validation target (see text), whereas the two grey circles illustrate one that does not.

Using the two-stage plan illustrated in Fig 1, our broadscale objective is to use results from virtual experiments employing two different systems to help explain IVIVE discrepancies. One system is a discretized software analog of an in vitro hepatocyte culture and the other is a software analog of a liver within a human subject. Concretized software analogs of hepatocytes are the key biomimetic components in both systems. The plan begins with a discrepant IVIVE prediction of hepatic clearance in humans. The Stage One goal is to obtain a direct quantitative mapping between temporal measures of simulated drug removal during virtual hepatocyte culture experiments and the data used to compute intrinsic clearance. The objective for Stage Two, which is independent of Stage One, is to achieve a direct quantitative mapping between temporal measures of drug removal during experiments using a virtual human and the data used to compute hepatic clearance. Hepatocyte objects can be identical in both systems. Simulated drug properties reflect target drug properties [5–9]. We achieve both objectives by adjusting parameters controlling the dynamics of drug interactions with hepatocytes and with other system components during execution. Once the objective for each Stage has been achieved, we observe and measure consequences of drug disposition and removal events at different locations within each system as executions unfold. We use those observations to develop and support a mechanism-based explanation for differences (or lack thereof) in disposition and removal details within the two systems and put forward a testable (falsifiable) theory: the actual mechanisms responsible for the observed IVIVE discrepancy and our model explanation are strongly analogous. Hereafter, to simplify descriptions, we distinguish virtual systems and components from real counterparts by appending the prefix “v,” and we distinguish “virtual” characteristics, properties, and phenomena from real counterparts with capitalization.

We construct vCultures and vHumans independently using the same objects, methods, and modular components. The structure of a vCulture is strongly analogous to that of a two-dimensional hepatocyte culture. A vHepatocyte (vHPC) object maps to actual hepatocytes in vitro and in vivo. Mobile vCompound objects represent the referent drug. The model mechanisms responsible for vCompound disposition and removal during vCulture and vHuman executions are intended to be strongly analogous to their in vitro and in vivo counterparts.

Recent reports document that earlier versions of vCulture and vHuman met demanding requirements. They also explain how model mechanisms were iteratively refined so that measurements made during virtual experiments mapped quantitatively to corresponding in vitro and in vivo measurements [8–14]. After achieving multiple validation targets, we argued that the model mechanisms responsible for vCompound disposition and removal, and the actual disposition and removal details within their referents, were strongly analogous at corresponding levels of granularity. For this work, we build on those arguments using improved versions of both systems.

Consider a particular IVIVE underprediction and assume that we have achieved Stages One and Two objectives. At that stage, measures of vCompound removal obtained during vCulture experiments will underpredict corresponding measures obtained during vHuman experiments. We can observe and measure temporal details as they unfold within both systems during execution and use them to develop a mechanism-based explanation for the virtual underprediction. That explanation can stand as a plausible theory explaining the targeted IVIVE underprediction, one that can be challenged and iteratively improved, as needed. To enable such theories to become credible, we must accumulate supporting evidence. This report provides the initial installment of that evidence.

A requisite for a successful Fig 1 plan is evidence documenting that, for a subset of vCompound properties, measures recorded during vCulture experiments directly predict corresponding measures made during vHuman experiments. A core postulate for IVIVE methods is that, under ideal conditions, including matched rates of drug delivery, there is a 1:1 equivalency between in vitro removal rate (intrinsic clearance) of unbound drug per hepatocyte (or microsomal equivalents) and the in vivo removal rate of unbound drug per hepatocyte. Although it is infeasible to obtain the latter measure in humans, it is feasible to do so during vHuman experiments. Hence, we use that 1:1 equivalency as a cross-system validation target [15]. We verify that, for a subset of vCompound properties, the equivalency is achieved (illustrated by area of overlap in Fig 1B). However, given that IVIVE methods frequently fail to adequately predict hepatic clearance, we are more interested in cases where measures made during vCulture experiments either over- or underpredict corresponding measures made during vHuman experiments (the non-overlapping areas in Fig 1B). We present several examples of discrepant predictions along with model mechanism-based explanations. The discrepancies are caused by differences in the dynamic exposure of vCompounds to vLiver vHPCs relative to vCulture vHPCs, which trace directly to vLiver-vCulture differences in the structural organization of vHPCs within the two systems. Because of those structural differences, vHPCs within the vLiver, compared to vCulture can on average be more efficient at removing vCompounds, *ceteris paribus*. The difference in vHPC efficiency is noteworthy because most in vitro-to-in vivo extrapolation methods for predicting hepatic clearance abstract away in vitro-in vivo differences in hepatocyte structural organization. Such observations support the idea that results of virtual experiments of the type illustrated in Fig 1 can help disentangle the mechanisms responsible for IVIVE discrepancies. Revising IVIVE methods to account for in vitro-to-in vivo differences in structural efficiency may be a strategy to reduce those discrepancies.

## Methods

### Model mechanisms and requirements that enable virtual experiments

We use previously validated agent-oriented, discrete-event methods. Model execution is a discrete-event Monte Carlo (MC) simulation. The virtual experiment approach involves building, experimenting on, and iteratively refining concrete model mechanisms [6, 7, 16], while seeking a balance between more detailed biomimicry and the increase in computation programmed into the model systems. Concrete model mechanisms differ fundamentally from the equation-based models [17]. During execution, model mechanisms generate phenomena that we hypothesize can become qualitatively and quantitatively similar to corresponding wet-lab measures. Once we meet the similarity criteria for both Fig 1 Stages, we can claim that vCulture and vHuman model mechanisms are strongly analogous to the real in vitro and in vivo mechanisms. To support that claim, the vCulture and vHuman systems must meet the following requirements.

1. They use absolute grounding [7]. The equation-based models employed by conventional IVIVE methods use absolute grounding, where variables, parameters, inputs, and outputs are in real-world units. Absolute grounding has important advantages and uses, but it limits model reuse and flexibility [5, 7, 17]. The Fig 1A plan anticipates that we will reuse the vCulture and vHuman systems without significant structural change. Model mechanisms employ relational grounding to deliver the flexibility required and enable model mechanism falsification. Relational grounding requires that variables, parameters, inputs, and outputs are in units defined by other components within each system. Hence, separate quantitative mapping models are required to relate virtual to actual measures. Such model separation increases flexibility and enables the required system reuse. Note that the vCulture–in vitro and vHuman-in vivo quantitative mapping models for different measures will be different necessarily. For clarity, parameter names are italicized.
2. Components and spaces are concrete and sufficiently biomimetic to facilitate analogical reasoning [18, 19].
3. Model mechanisms have context and exhibit the characteristics of an explanatory biological mechanism [20]. Fixed components (e.g., Cells) are arranged spatially and exhibit structure, localization, orientation, connectivity, and compartmentalization. vCompound dynamics mediated by the model mechanisms have temporal aspects, including rate, order, and duration.
4. A vCompound type can map to a particular chemical entity. During each time-step, quasi-autonomous components (i.e., software agents such as Sinusoidal Segments (SSs) and vHPCs) recognize different vCompound types (elaborated below) and adjust their responses appropriately. For example, a vHPC recognizes that an adjacent vCompound has the property *membraneCrossing* = yes and allows it to Enter (not Enter) stochastically, when other conditions (if any) are met.

**Fig 2:**
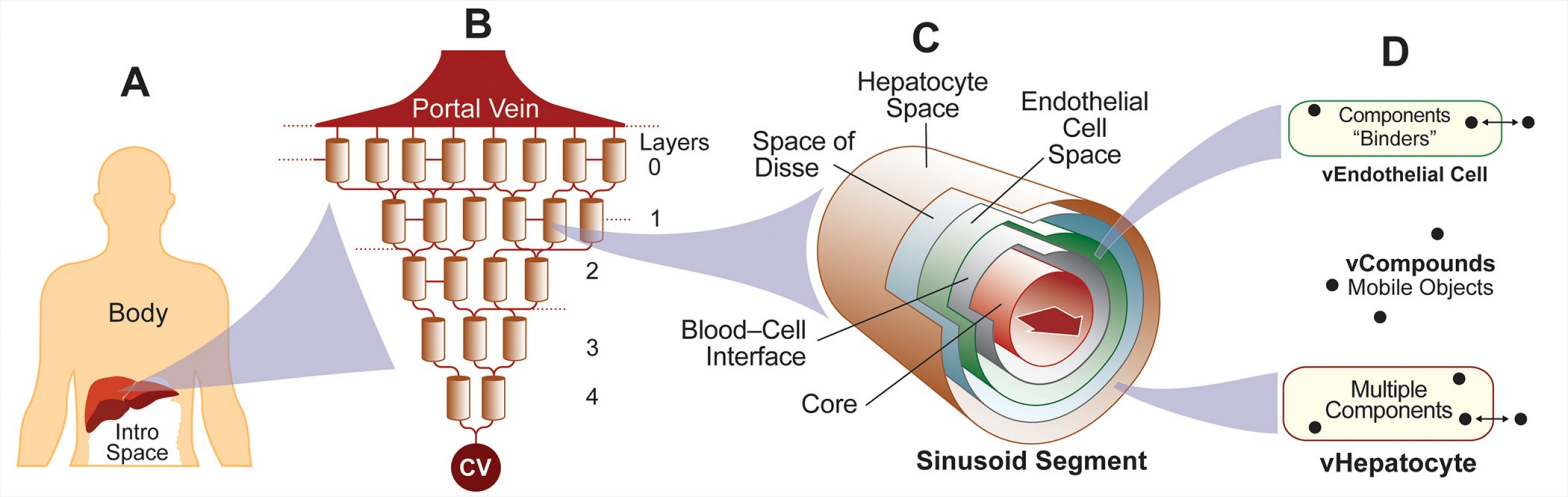
Virtual Human components. (A) A vHuman comprises a well-mixed Body space, a vLiver, and a space for Dose. (B) A portion of vLiver. Graph edges designate flow connections within and between Layers. (C) A multi-layered, quasi-3D Sinusoidal Segment maps to a portion of lobular tissue. It comprises a Core surrounded concentrically by the four 2D grids described in the text. Mobile vCompound objects move within and between these grids. (D) A Sinusoidal Segment contains two Cell types. Each type controls vCompound entry from and exit to an adjacent space. The Cell and the components within determine the fate of vCompounds that enter. We list specification details in S1 Table.

Further, we make four claims and cite supporting evidence. 1) The parsimonious structural organization of vHPCs within the vLiver (Fig 2) is sufficiently analogous to the histological organization of hepatocytes within human and rodent livers [21, 22]. Use-case-specific validation evidence is provided in [12, 14, 23]. 2) The fine-grain model mechanisms responsible for the removal of vCompounds within vHPCs are concrete, biomimetic, and can be parameterized to be strongly analogous to counterparts in vivo and in vitro. Supportive, use-case-specific evidence is provided in five reports [8, 10–12, 14]. 3) The structural organization of vHPCs within vCultures is sufficiently biomimetic to simulate the in vitro experiments and measures used to compute intrinsic clearance [8, 10, 11]. 4) When averaged over many Monte Carlo-sampled executions, mean measures of vCompound dynamics can be scaled to match corresponding wet-lab measurements within prespecified quantitative criteria. Use-case-specific validation evidence is provided in three reports [10, 12, 14].

### The cross-system validation target

Each vCompound type is assigned a unique set of properties (parameterizations, described below). The plan in Fig 1A requires that, for a subset of vCompound types, the core postulate for IVIVE methods, mentioned above, must be valid. Specifically, measures of the per vHPC removal rates of unbound vCompounds made during independent vCulture and vHuman experiments will be quantitatively equivalent within some similarity criterion, *ceteris paribus*. That equivalency, illustrated by area of overlap in Fig 1B, serves as the cross-system validation target [15] for this work.

Each time an unbound vCompound enters a particular vHPC, we record that event. A vCompound’s fate after entering a vHPC is independent of the system’s structure and vHPC locations within. Absent a mechanism that enables a vHPC to “actively” internalize a bound vCompound, vCompound Entry and removal rates are directly correlated. Demonstrating quantitative equivalency of vCompound Entry rates per vHPC is also evidence that we have achieved the cross-system validation target. The two grey circles in Fig 1B illustrate a vCompound type that does not achieve the cross-system validation target. Consequently, measures of its Entry and removal rates per vHPC will either under- or overpredict corresponding vHuman experiment measures.

### Simulation time and vCompounds

During virtual experiments, time advances in discrete time-steps (TS; also called simulation cycles). The duration of each execution is 21,600 TS. For this work, mappings of TS to real time units is required. In recent work employing virtual acetaminophen hepatotoxicity experiments, the referent organisms are mice [12] and one TS maps to one second. Events occur at a particular instant in time, marking a change of system state. Measurements made at the end of each TS may map to corresponding referent measurements (real or envisioned). However, within a TS, some execution events are codebase dependent and have no direct wet-lab counterparts. We intend that state changes at the conclusion of a TS will be analogous to the net consequences of fine-grain processes that occur in parallel within the referent during the corresponding time interval. To simulate that parallelism, the order of events is randomized for each TS.

We study four vCompound types (vC1-vC4) and Marker. Later, we describe their behaviors during experiments (**vCompound dynamics during experiments**). The Dose for each experiment (vHuman and vCulture) is 100,000 objects. Marker, which is always 50% of Dose, serves as a multi-attribute virtual internal standard. Marker does not enter Cells (parameter *membraneCrossing* = false). For vC1-vC4, *membraneCrossing* = true. For this work, only fluid mechanics properties are simulated. All other properties, including those that would map directly to the physicochemical properties of real compounds, are absent. They were not needed, but they can be added in later works, as needed. Mean Marker behavior during repeat executions of the same system is the same, within the variance of MC-sampled executions. As explained in Smith et al., Marker is particularly efficacious during cross-system validation experiments and verification following code changes [12].

vC1-vC4 mimic high permeability xenobiotics. We had three reasons for limiting attention to only highly permeable vCompounds. 1) We expected that the behaviors of highly permeable vCompounds would make it straightforward to identify vCompound types that achieve the validation target, and 2) make it easier to detect, identify, and correct any inadvertent non-biomimetic differences (having no wet-lab counterparts) between the two systems. 3) By doing so, the duration of executions is reduced. Subsequent work will be needed to establish vHuman-vCulture cross-model verifications for less permeable vCompounds.

### vHuman and vLiver components

Upon initiating an execution, all vHuman and vCulture components are created, assembled, and parameterized. We then initiate the experiment protocol and begin measurements. One experiment is a fixed number of MC executions, with a different pseudo-random number seed for each execution. Because all events are stochastic, the variance of TS-to-TS measures of the same event type (e.g., mean entry rate per vHPC and Extraction Ratio) during one execution can be large (as illustrated in [11]). Consequently, we average those measures over some number MC executions. We specify a number that is sufficient to detect (by visual inspection) significant changes in measured phenomena caused by parameter differences between experiments. As in recent work employing similar vLivers [11, 12], averaging measures over 12 MC executions proved to be sufficient for this work.

A vHuman (Fig 2A) comprises a vLiver (detailed below), Body, and an Intro space to contain Dose. A vLiver plugs together quasi-autonomous software objects that represent hepatic components at different scales and levels of detail. Microarchitectural features are represented separately from the mechanisms that influence vCompound disposition and Metabolism. A vLiver = 12 MC-sampled vLobules. One vLobule maps to a small random sample of possible lobular flow paths within a whole liver along with all associated hepatic tissue. It is a rough analogy of an actual mammalian lobule, but components are organized to mimic the 3D organization of tissue within actual lobules [8, 21]. A directed acyclic graph, with a Sinusoidal Segment (SS) object at each graph node, mediates flow within a vLobule. Flow follows the directed graph edges connecting SSs. Flow paths map to averages of actual flow paths within hepatic sinusoids.

**Fig 3:**
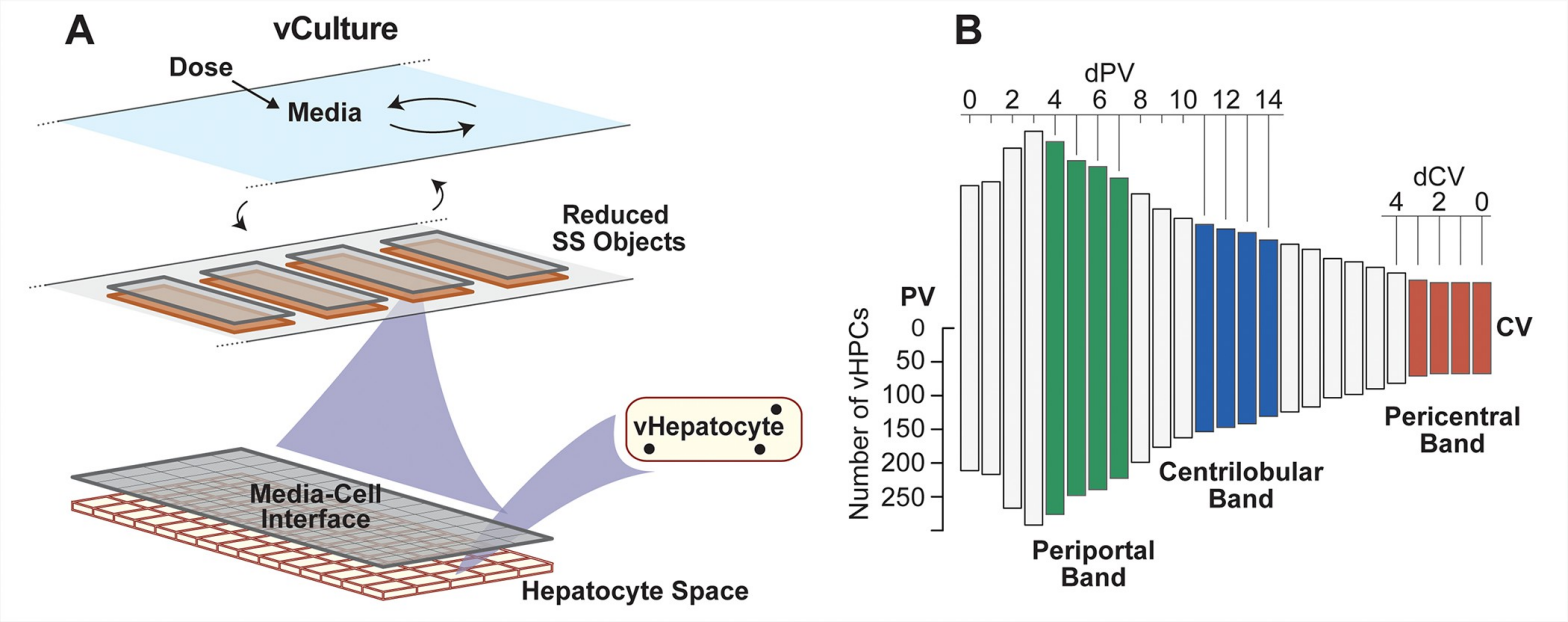
vCulture configuration and relative PV-to-CV vHPC density within vLobules. (A) A vCulture comprises a Media Space plus a single Layer of same-size SS objects (reduced relative to those in Fig 2C). The well-mixed Media Space functions the same as Body. Specification details are listed in S1 Table. (B) Each bar’s height represents the mean number of vHPCs at the indicated vLobule location averaged over 12 Monte Carlo executions. Moving left-to-right from PV (dPV), the first 15 bars correspond to locations at increasing distances from PV along the average PV-to-CV path. Moving right-to-left, the first 10 bars correspond to locations at increasing distances from CV along the average CV-to-PV path. We average measures within the Periportal, Centrilobular, and Pericentral bands to characterize PV-to-CV differences in vCompound Entry and removal rates per vHPC.

Quasi-3D SS objects are software agents. Each one comprises a Core and five 2D grids arranged concentrically: Blood-Cell Interface Space (simply Interface Space hereafter), Endothelial Cell Space, Space of Disse, Hepatocyte Space, and Bile space (not used in this work and not shown in Fig 2A). An SS in a particular vLobule Layer (described below) functions as an analog of sinusoid components and features at corresponding relative locations averaged across many actual lobules. SS dimensions are MC-sampled, within constraints, at the start of each experiment to mimic lobular variability and simulate a wide variety of Periportal (PP) to Pericentral (PC) flow paths. To minimize differences in vCompound dynamics between vCulture and vHuman experiments, we tightened the constraints on SS dimensions used by Smith et al. [12, 14] so that mean SS dimensions in vLivers closely matched the Hepatocyte Space (Fig 3A) dimensions used during vCulture experiments (see **vCulture components**). Thus, SS width is clamped at 15 grid spaces, and the mean length is approximately 5 grid spaces. The mean minimum (maximum) SS length is 3.2 (7.3) grid spaces.

A vLobule has five layers, which can map to lobular zones (Fig 2B). The Portal Vein (PV) connects to all Layer 0 SSs. From the PV to the Central Vein (CV), there are 45, 25, 20, 15, and 9 SS per Layer. There are 55 Layer 0-to-1 edges; 65 Layer 1-to-2; 35 Layer 2-to-3, and 25 Layer 3-to-4 edges. A graph edge connects each Layer 4 SS to the CV. There are also intra-Layer edges (randomly assigned for each execution), which mimic connections between sinusoids: 20 within Layer 0, 7 within Layer 1, 5 within Layer 2, 2 within Layer 3, but none within Layer 4. We fix numbers of intra- and inter-Layer edges for all experiments, but their SS-to-SS connections are Monte Carlo-sampled for each execution. Having more edges than SSs enables mimicking the wide variety of PV-to-CV flow paths within lobules [24]. Interconnections are essential to enable the same vLiver to achieve previously described isolated perfused liver validation targets for several different drugs [13, 14]. The mean number of vHPCs per vHuman execution = 8,475 (SD = 167). Fig 3B illustrates the mean number of vHPCs at different PV-to-CV locations.

We started with the 3-Layer graph structure used by Smith et al. [12] and increased Layers (from three to five), number of SSs (from 68 to 144), number of graph edges, and reduced mean SS circumferences and lengths by approximately 50%, while maintaining carrying capacity. Those changes improved hepatic disposition biomimicry; specifically, they improved vCompound lateral dispersion and reduced the parallel nature of SS flow paths in Layer 4. The vLobule’s structure was achieved after following the Iterative Refinement Protocol [14, 25, 26] to achieve qualitative and quantitative cross-validation measures of Marker dynamics and acetaminophen hepatotoxicity measures before and after the above upgrades.

Cell objects are software agents. During each TS, they mediate all interactions with vCompounds, including entry and exit, based on vCompound type properties. Endothelial Cells occupy 99% Endothelial Cell Space and vHPCs occupy 100% of Hepatocyte Space. Other Cell types can be included when required, as in Petersen et al. [10]. Both Endothelial Cells and vHPCs contain binder objects to simulate non-specific binding. They map to a conflation of all cell components responsible for non-specific binding of the referent drug.

In part because we use relational rather than absolute grounding, a vHPC object does not map 1:1 to a hepatocyte (explained in **Component biomimicry has limits**). Events occurring within vHPCs (discussed below) are intended to map directly to corresponding events believed to occur within hepatocytes, as in Smith et al. [12].

### vCulture components

vCulture is a partially deconstructed variant of a vHuman. To support achieving the cross-system validation target, we revised the codebases used by Smith et al. to minimize the differences between vCulture and vHuman codebases [12]. Intro space is retained. The well-mixed Body space is retained but renamed Media space. We collapse the directed graph in Fig 2B to a single Layer 0 having 144 nodes, which is invariant over MC executions. vCulture’s single Layer uses 114 SS, which is the same as the number of SSs per vLobule. The Core, Endothelial Cell Space, and the Space of Disse in Fig 2C are not used. We merge Interface Space, PV, and CV to function as the Media-Cell Interface Space (Fig 3A). It is the same size as Hepatocyte Space. Media-Cell Interface can map to the unstirred water layer [27]. Each of the 144 Hepatocyte Spaces maps to some number of confluent hepatocytes within a hepatocyte culture. For simplicity, all Hepatocyte Spaces are 15 (w) x 5 (l) grid spaces, with one vHPC assigned to each, for total of 8,550 vHPCs, 75 more than the 8,475 in vHuman experiments, a difference of 0.885%.

### vCompound dynamics during experiments

Because vHPC numbers are essentially the same within both systems, we avoid scaling and make direct graphical comparisons of vCulture and vHuman measures when Dosing in both systems is configured the same, which is the case for all experiments described below. However, when required by Stage One or Two, Dosing can be altered. Upon execution, both systems add Dose to the Intro space. Each TS after that, a fraction of Dose in Intro space is transferred to Body (Media), simulating first order absorption (first order addition to Media). For convenience, we reused the simulated absorption rate used previously for acetaminophen [14].

vCompounds enter the vLiver from Body via the PV. A fraction of vCompounds in Body is moved to the PV each TS to mimic hepatic blood flow. Each TS, vCompounds in the PV are moved randomly to the Core or Interface Spaces in Layer-0 SSs. The parameter *ssFlowRate* controls simulated blood flow in the Core. vCompounds exit an SS via Core and Interface Space and are moved to a lateral or downstream SS along a randomly selected connecting graph edge. Within an SS, extra-Cellular vCompounds percolate stochastically through accessible extra-Cellular spaces influenced by three local flow parameters. Outside the Core, extra-Cellular movement is a biased random walk controlled by the values of *forwardBias* and *lateralBias* listed in S1 Table. Those values are the same for Marker and vC1-vC4; however, they can be vCompound-specific. vCompounds that exit a Layer-4 SS to the CV are returned to Body. Measurements of vCompound in Body can map quantitatively to measures of a referent drug in plasma (or blood). Because we measure vCompound amounts entering and exiting vLiver each TS, we can compute vLiver Extraction Ratio each TS analogous to how it is calculated during perfused liver experiments.

vLiver Extraction Ratio = (vCompounds_PV_−vCompounds_CV_) / vCompounds_CV_

Each TS during a vCulture experiment, a fraction of vCompounds in Media are transferred to Media-Cell Interface. vCompounds movements within and between Media-Cell Interface and Hepatocyte Space are the same as within and between the Space of Disse and Hepatocyte Space within SSs. vCompounds exit the Media-Cell Interface and return to Media. Because we measure vCompound amounts entering and exiting Media-Cell Interface each TS, we can compute a vCulture Extraction Ratio each TS.

vCulture Extraction Ratio = (amount entering–amount exiting Interface Space) / amount entering Interface Space.

### Focusing on vCompound-vHPC Entry events

vHPCs, their components, and the rules governing component interactions with vCompounds are the same in both systems, but they can be customized as needed during Stages 1 and 2. In this work, the fate of a vCompound after it enters a vHPC is independent of the system structure and vHPC locations within the system. Each TS, a vCompound that is collocated with a vHPC is allowed to Enter (or not Enter) randomly. An Entry event is a requisite for Metabolism and Removal. The value of the vCompound-specific probabilistic parameter *pEnter* determines the occurrence of a vCompound-vHPC Entry event (simply Entry event hereafter). Structural differences between vLivers and vCultures can influence the probability each TS of a vCompound being collocated with a vHPC and thus the occurrence of an Entry event.

We record the number of Entry events for each vHPC each TS. Comparing mean Entry rates per vHPC during vCulture and vHuman experiments provides the most reliable method to directly determine the equivalency (or lack thereof) between vCompound disposition and removal dynamics within the two systems. Hereafter, we rely on equivalency of Entry rates per vHPC (hereafter, simply Entry rates) as the cross-system validation target. We also use Entry rate differences to help explain differences in other measures that have wet-lab counterparts, such as the temporal profiles for removal of vCompound from Media and Body.

IVIVE methods typically assume that only the unbound xenobiotic adjacent to a hepatocyte is available to enter. For this work, the probability of a vCompound Entry event (*pEnter*) = (fraction_unbound)×(pEnter_unbound), where pEnter_unbound is the probability of an Entry event for an unbound vCompound. Because the vCompounds in this work represent highly permeable xenobiotics, for simplicity we specify that pEnter_unbound = 1. Thus, hereafter, *pEnter* = fraction_unbound. Each TS, an unbound intra-Cellular vCompound is allowed to exit randomly. The vCompound-specific parameter *pExit* determines the subsequent occurrence of an Exit event.

Under identical dosing conditions, vCulture-vLiver differences in extra-vHPC events and in the ability of the vCompound to Enter (Exit) vHPCs may break the 1:1 equivalency and prevent achieving the cross-system validation target. For the experiments that follow, we focus on the degree to which changing *pEnter* may break the 1:1 equivalency.

vC1 and vC2 represent the hypothetical extremes for removing highly permeable xenobiotics. vC1 is not removed; it simply enters and exits all vHPCs. *pExit* for vC1 = 1. vC2 experiences maximal removal; it enters but does not exit vHPCs (*pExit* = 0). vC3 mimics xenobiotics having a small, near zero hepatic extraction ratio. vC4 mimics xenobiotics having a medium hepatic extraction ratio. For vC3 and vC4, *pExit* = 1.

Entry events exhibit no significant location dependency during vCulture experiments. To characterize location-dependent differences in vHPC Entry events within a vLiver, we average measures over 12 MC executions within the PP, Centrilobular (CL), and PC bands illustrated in Fig 3B.

Hepatic clearance is defined as the volume of blood that is cleared of drug by the liver per unit of time. By design [7], there is no software counterpart to volume of blood (see **Component biomimicry has limits**). The virtual counterpart to hepatic clearance is the removal rate from Body. Because we employ relational grounding, a separate quantitative mapping is needed to convert the amount of vCompound removed per TS to the volume cleared of an amount of drug per unit of time, as in Petersen et al. [10].

### vCompound Metabolism

Following an Entry event, vC3 or vC4 may be Metabolized. Each vHPC contains Binder objects and four physiomimetic modules [8] that manage events involving Binders: *BindingHandler*, *MetabolismHandler*, *InductionHandler*, and *EliminationHandler*. The last two are not used in this work. An Enzyme object is a subtype of Binder objects. An Enzyme can bind and may Metabolize a bound vCompound. In this work, all Binders in vHPCs function as Enzymes. The number of Enzymes in each vHPC is subject to a random draw from *U*(*bindersPerCellMin*, *bindersPerCellMax*), where *bindersPerCellMin* = 5 and *bindersPerCellMax* = 10. Each TS, an unbound vCompound is given an opportunity, determined randomly, to bind to one unoccupied Enzyme. The value of the parameter *pBind* determines whether binding occurs. Upon binding, the vCompound is scheduled to be Metabolized, with probability *pMetabolize*, or released after *bindCycles* = 10 TS. A Metabolized vCompound is deleted and replaced by a Metabolite object. Each TS, a Metabolite is given an opportunity to exit randomly. The subsequent occurrence of an Exit event is determined by its value of *pExit*. *pExit* for vC3 and vC4 Metabolites = 1; however, when needed, *pExit* can be made Metabolite-specific. After exiting a vHPC, a Metabolite does not enter Cells. To facilitate direct comparisons of results of experiments using different vCompound types, the parameters controlling Metabolite movement within and between extra-Cellular spaces in both systems are the same as for vC3, vC4, and Marker. When needed, we can employ multiple Enzyme types [12, 14]. In those cases, *pBind*, *bindCycles*, *pMetabolize*, and *U*(*bindersPerCellMin*, *bindersPerCellMax*) can be Enzyme-type and vCompound-type-specific.

### Making virtual measurements analogous to wet-lab measurements

We measure virtual features and phenomena analogous to how corresponding wet-lab measurements are (or might be) made. Doing so strengthens the virtual-to-wet-lab experiment analogy. Many parameters are probabilistic. Specifications of several features are MC-sampled at the start of each execution. Because we average measurements over 12 MC executions, some may exhibit considerable variability, as described above. That variability is intentional. It represents and helps account for the variability and uncertainty that characterizes wet-lab measurements.

### Component biomimicry has limits

None of the objects in Figs 2 and 3A are intended to model actual biological counterparts explicitly. Instead, their organization, function, and behaviors during execution—the model mechanisms—are intended to be sufficiently analogous to their biological counterparts so that prespecified fine- and coarse-grain measures, recorded during executions can map quantitatively to corresponding Stage 2 and 2 validation targets [17].

An SS does not map directly to a portion of a single sinusoid and adjacent tissue. Instead, events occurring within a particular SS are intended to be strongly analogous to corresponding events occurring at corresponding relative PV-to-CV locations. The mapping from cylindrical 2D Hepatocyte Space in Fig 2C to corresponding 3D configurations of hepatocytes is an approximation. A vHPC at a particular PV-to-CV location within a vLiver maps to a random sample of hepatocytes (or hepatocyte functionality) accessed by referent drug at a corresponding relative PV-to-CV location. Ideally, each vHPC within a vCulture maps to a same-size random sample of hepatocytes (or hepatocyte functionality) isolated from a referent liver. Thus, a vHPC cannot map 1:1 to a hepatocyte, although it functional analogies are strong. It follows that a vLobule does not directly model liver microanatomy, yet its contribution to model mechanisms is intended to be hepato-mimetic during execution.

### Hardware and software details

The Java-based MASON multi-agent toolkit was used to develop the vLiver and vCulture. Experiments were executed using local hardware running 64-bit Linux Mint and Google compute engine was used as the virtual machine. The virtual framework was created using Java. The R programming language was used for analyses and plotting data. vHumans, vCultures, and configuration files are managed using the Subversion version control tool in two repositories, one private (Assembla) and another public. Values for key vHPC specifications and parameterizations are listed in S1 Table. Quality assurance and control details, along with practices followed for validation, verification, sensitivity analyses, and uncertainty quantification, areas discussed in Smith et al. [14]. The toolchain, operating system, configurations, and our entire codebase is available on (https://simtk.org/projects/isl/).

## Results

All results from experiments using vC1 (first subsection below) achieve the cross-system validation target. Results from experiments using vC2 achieve the cross-system validation target for *pEnter* = 1 (second subsection) but do not do so for *pEnter* ≤ 0.8. We provide mechanism-based explanations for those shortfalls. vC3 (third subsection) achieves the cross-system validation target for all values of *pEnter*. In the final subsection, we describe how and why vC4 does not achieve the cross-system validation target for all values of *pEnter*.

### vCompound-1 achieves the cross-system validation target

**Fig 4:**
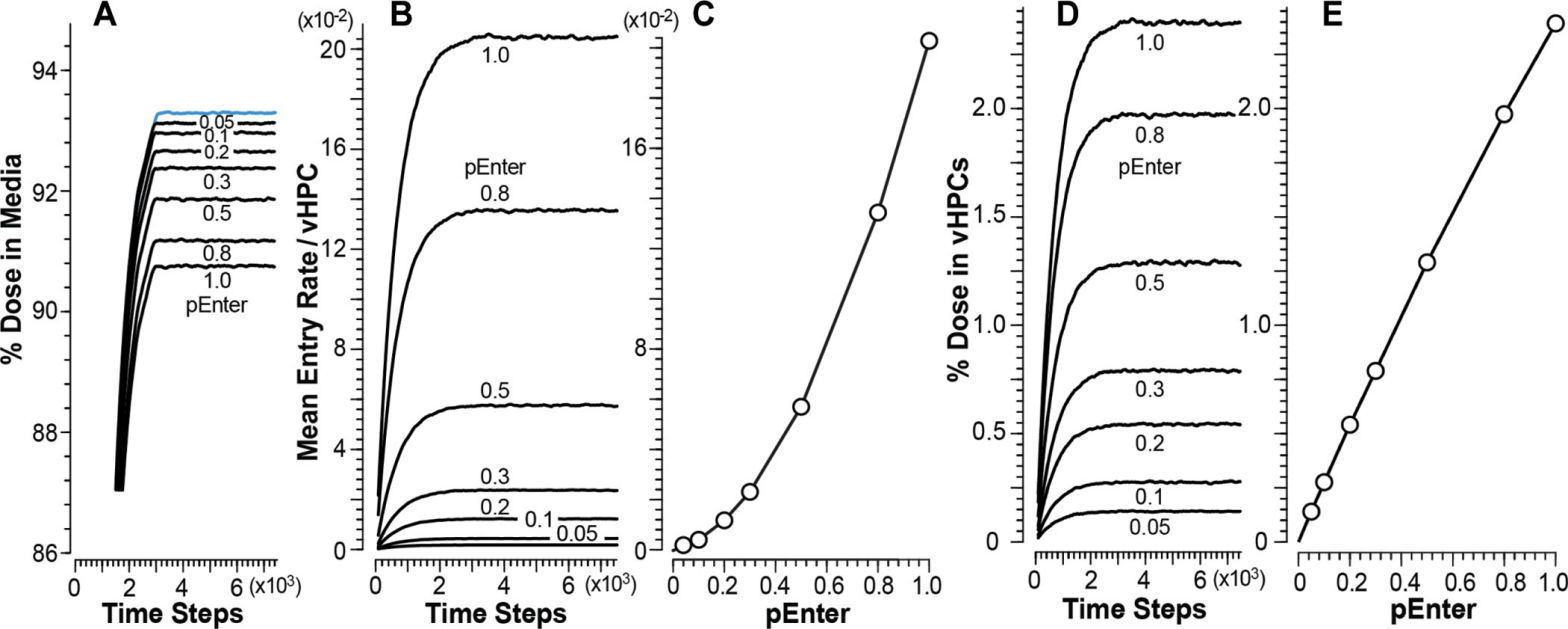
Results from vCulture experiments using vC1. Changing *pEnter* alters vCompound dynamics during each experiment. Temporal values here, and in the subsequent figures, are centered moving averages spanning 181 TS. (A) Temporal measures of percent Dose in Media for each *pEnter*; blue measures are for Marker. (B) Temporal measures of mean Entry rates. (C) Mean dynamic steady-state Entry rates for each *pEnter*. (D) Temporal measures of percent Dose and (E) mean dynamic steady-state values of percent Dose within all vHPCs for each *pEnter*.

**Fig 5:**
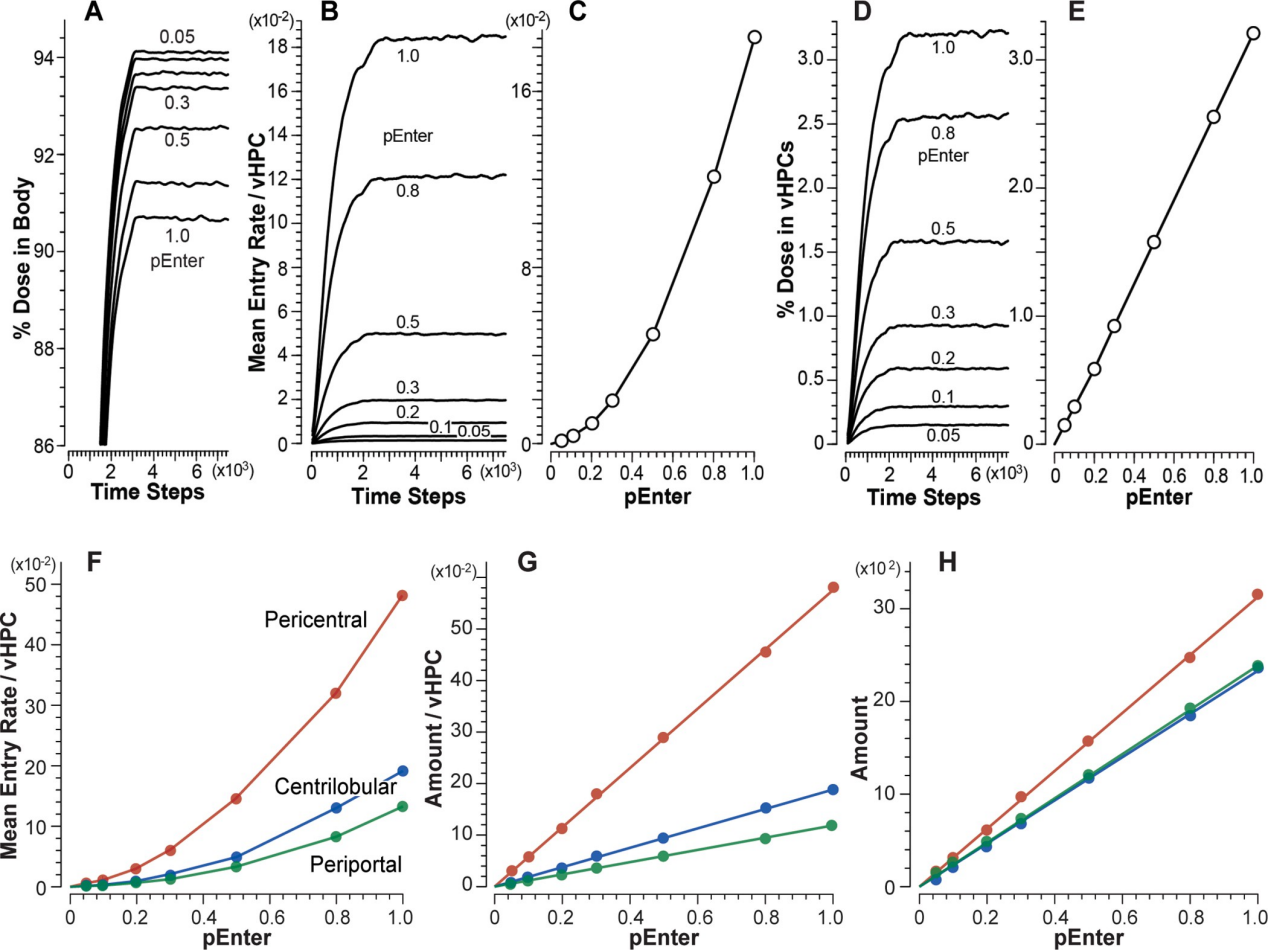
Results from vHuman experiments using vC1. (A) Temporal measures of percent Dose in Body for each *pEnter*. (B) Temporal measures of mean Entry rates for each *pEnter*. (C) Correlation between mean dynamic steady-state Entry rates and *pEnter*. (D) Temporal measures of percent Dose in vHPCs for each *pEnter*. (E) Correlation between mean dynamic steady-state amounts of vC1 in vHPCs and *pEnter*. Measures in F-H are mean dynamic steady-state values within the Periportal, Centrilobular, and Pericentral bands in Fig 3B. (F) Correlation between mean dynamic steady-state Entry rates and *pEnter*. (G) Correlation between vC1 amounts per vHPC and (H) total amounts within all vHPC and *pEnter*.

Figs 4 and 5 contain mean measures of vC1 during vCulture and vHuman experiments. Under dynamic steady-state conditions, for *pEnter* = 1.0, the mean plateau values for percent of Dose in Media (Fig 4A) and vHuman Body (Fig 5A) are equivalent within the variance of 12 MC executions. The pattern of changing plateau values is similar when using smaller *pEnter* values. However, for larger *pEnter* values, the vCulture plateau values are smaller than corresponding values from vHuman experiments.

The mean plateau Entry rates in Figs 4B and 5B decrease with decreasing values of *pEnter*. For each *pEnter*, the mean Entry rates are smaller in vLiver (most evident for *pEnter* = 1.0) because a small fraction of vC1 within Core, Interface Space, Endothelial Cell Space, and the Space of Disse exits to CV without accessing Hepatocyte Space. Hence, the accessibility of those additional spaces reduces slightly the dynamic exposure of vC1 to vLiver vHPCs relative to vCulture vHPCs. A vCompound is exposed to a vHPC when it is collocated with a vHPC during a simulation cycle. After considering exposure difference and the variance across 12 MC executions, mean plateau vC1 Entry rates for all *pEnter* values are equivalent in both systems. Thus, we achieve the cross-system validation target, and vCulture Entry rates adequately predict corresponding vHuman Entry rates for all *pEnter*.

The correlations between Entry rates and *pEnter* (Figs 4C and 5C) exhibit the same nonlinearity. After entering vCulture’s Media-Cell Interface Space (or the Space of Disse within a vLiver SS), a vC1 using *pEnter* = 0.5–1.0 may enter and exit more than one vHPC before exiting to Media (vCulture) or exiting that SS and entering a downstream SS (vLiver). In both cases, the probability of multiple Entry events is reduced considerably for vC1s using smaller *pEnter* values. Under dynamic steady-state conditions, we expect vC1 amount per vHPC to be directly proportional to *pEnter*. Correlations between amounts in vHPCs and *pEnter* (Figs 4E and 5E) verify that expectation.

Mean plateau vC1 Entry rates and amounts per vHPC are the same for all vCulture vHPCs. That is not the case for vHuman experiments (Fig 5G, 5H), where measures depend on the vHPC’s PV-to-CV location. Because the number of vHPCs decreases PV-to-CV (Fig 3B), the correlation between mean Entry rates and *pEnter* (Fig 5F) and between mean amounts per vHPC and *pEnter* (Fig 5G) is largest within the PC band and smallest within the PP band. For *pEnter* = 1.0, mean plateau Entry rates increase 1.4-fold (3.6-fold) from the PP to the CL (PC) band. The increase is larger for smaller *pEnter* values. For example, when using *pEnter* = 0.1, mean Entry rates increased 1.7-fold (5-fold) from the PP to the CL (PC) band. The explanation for that nonlinear pattern is the same as that provided above for Figs 4C and 5C. Although the amount per vHPC is larger within the CL band than within the PP band, there are fewer vHPC within the CL band. Consequently, mean amounts within the two bands are similar (Fig 5H). In similar experiments that otherwise have a constant PV-to-CV vHPC density (analogous to the conventional parallel tube liver model), mean Entry rates and amount of vC1 per vHPC would decrease PP-to-CL-to-PC.

### Achieving and not achieving the cross-system validation target using vCompound-2

**Fig 6:**
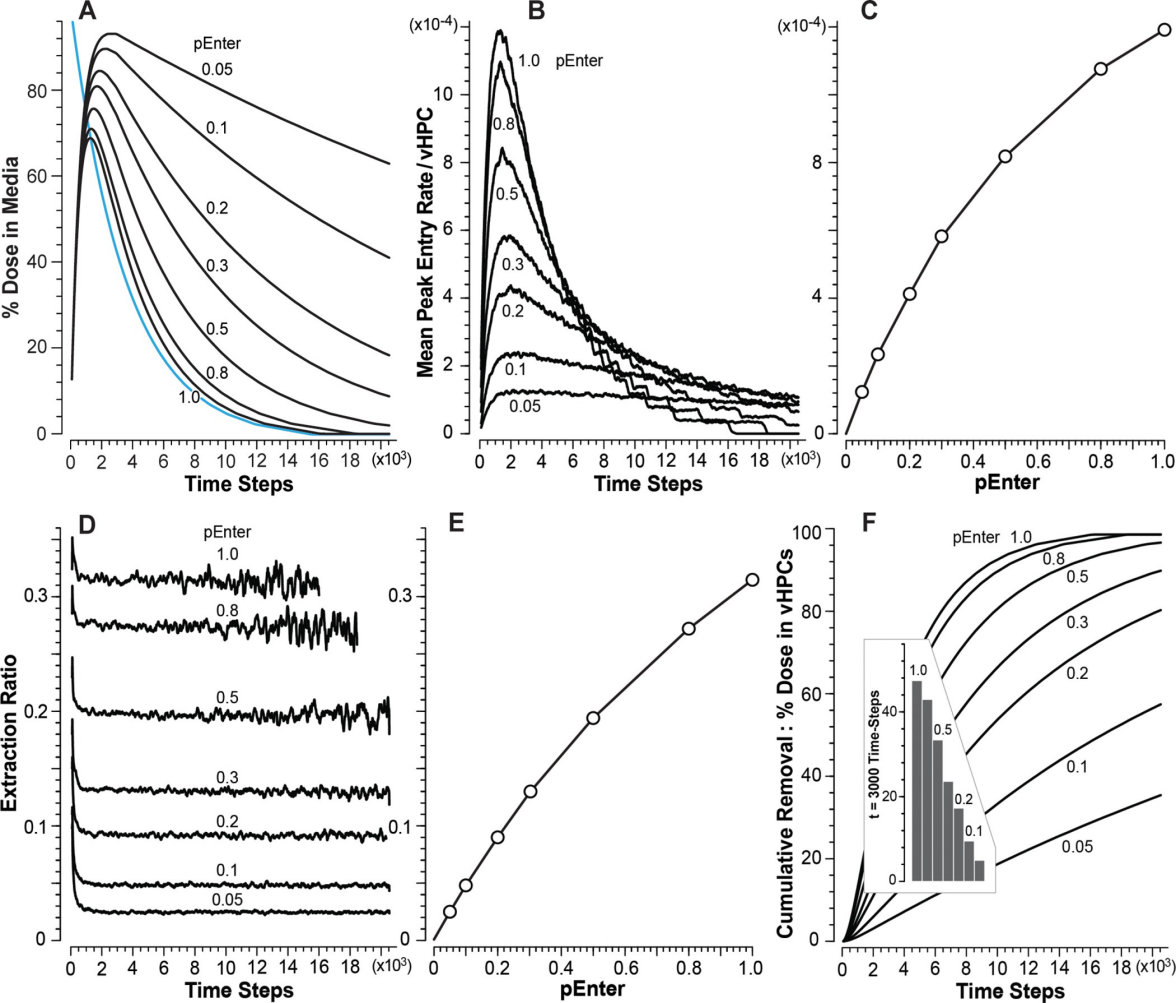
Results from vCulture experiments using vC2. (A) Temporal measures of percent Dose in Media for each *pEnter*. Blue profile: added all vC2 to Media at t = 0. (B) Mean removal rates for each *pEnter* (Entry rates = removal rates). (C) Correlation between mean peak Entry rates and *pEnter*. (D) Temporal measures of Extraction Ratio for each *pEnter*. Average variance increases with time because smaller amounts are measured each TS. Extraction Ratios for *pEnter* = 1.0 and 0.8 terminate because (essentially) all vC2 has been removed. (E) Correlation between mean plateau Extraction Ratios and *pEnter*. (F) Temporal measures of cumulative percent Dose in vHPCs for each *pEnter*. Insert: mean values at t = 3,000 TS for each *pEnter*.

**Fig 7:**
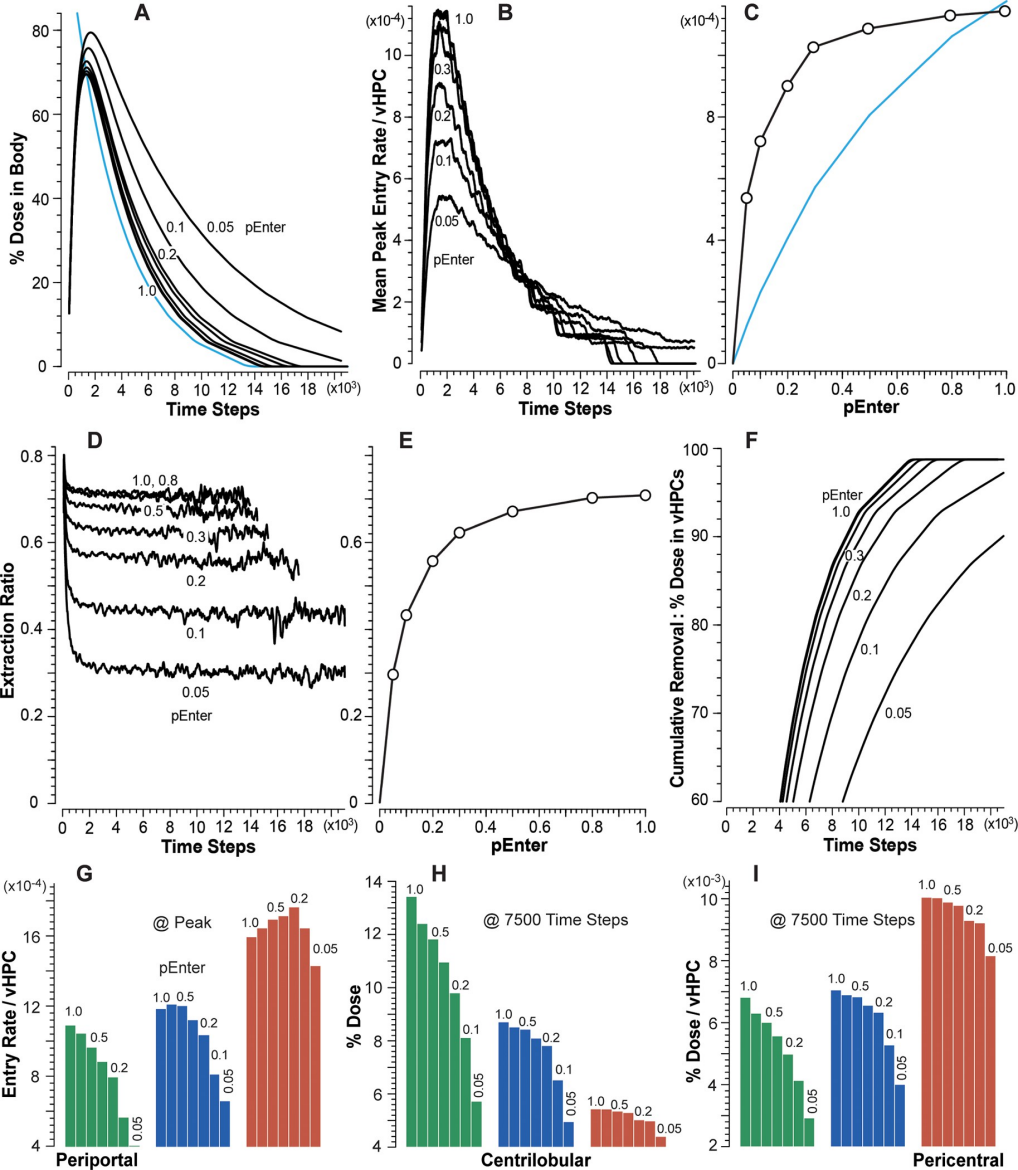
Results from vHuman experiments using vC2. (A) Temporal measures of percent Dose in Body for each *pEnter*. Blue profile: all vC2 was added to Media at t = 0. (B) Mean Entry rates for each *pEnter* (Entry rates = removal rates). (C) Correlation between mean peak Entry rates and *pEnter*. To facilitate comparisons, the blue curve traces the corresponding values from Fig 6C. (D) Temporal measures of Extraction Ratio for each *pEnter*. For p ≥ 0.2, measures terminate early because (essentially) all vC2 has been removed. Variance increases with time because smaller amounts are measured. (E) Correlation between mean plateau Extraction Ratios and *pEnter*. (F) Temporal measures of cumulative percent Dose in vLiver for each *pEnter*. Note that, compared to Fig 6F, vLiver removal of vC2 is strikingly more efficient. (G) Peak Entry rates for each *pEnter* within the three bands in Fig 3A. Corresponding measures of percent Dose (H) and percent Dose per vHPC (I) at 7500 TS.

Figs 6 and 7 show measures of vC2 disposition and removal during vCulture and vHuman experiments. Because *pExit* = 0, a vC2 Entry event is also a removal event. After taking into account that Interface Space, Endothelial Cell Space, and the Space of Disse are absent in vCultures, the temporal profiles (and areas under each curve) for percent dose in Media (Fig 6A) and Body (Fig 7A) are equivalent for *pEnter* = 1.0 within the variance of 12 MC executions. The Entry rate profiles (Figs 6B and 7B) for *pEnter* = 1.0 are also equivalent. Thus, for *pEnter* = 1.0, the cross-system validation target is achieved: vCulture measures predict corresponding vHuman values. However, for *pEnter* = ≤ 0.8, the cross-system validation target is not achieved: vCulture measures underpredict corresponding vHuman measures and the magnitude of the underprediction increases as *pEnter* decreases.

During vCulture experiments, the correlation between mean peak vC2 Entry rates (averaged over 1,000 TS) and *pEnter* (Fig 6C) is concave. Conventional theory is that it should be linear. The Entry rates for *pEnter* ≤ 0.8 are larger than one might expect. The reason for the nonlinearity: when a vC2 (using *pEnter* ≤ 0.8) adjacent to a vHPC in Hepatocyte Space is not allowed to enter, when given an opportunity, it may have one or more additional opportunities to enter a vHPC before returning to Media. The net consequence of those stochastic events is analogous to the unstirred water layer effect described by Wood et al. [27]. The nonlinearity in Fig 6C is also evident in percent Dose remaining in Media (Fig 6A), Extraction Ratios (Fig 6D and 6E), and cumulative removal (Fig 6F).

When using *pEnter* ≤ 0.8, measures of percent Dose in Media (Fig 6A) and cumulative removal (Fig 6F) during vCulture experiments underpredict corresponding measures during vHuman experiments. It is noteworthy that measures in Fig 7A–7D and 7F are relatively robust to changes in *pEnter* within the 0.5–1.0 range. For vCulture experiments using *pEnter* = ≤ 0.5, mean peak Entry rates (and areas under percent Dose in Media curves) considerably underpredict corresponding vHuman measures (Fig 7B). An example for *pEnter* = 0.3 (0.1; 0.05), the mean peak Entry rate is underpredicted by 1.4-fold (3.1- and 4.4-fold). Those underpredictions are a consequence of the tapered structural organization of vHPCs within vLivers (Fig 2B) being absent in vCultures. The fine-grain events occurring within PP, CL, and PC bands explain how and why measures made during vCulture experiments underpredict corresponding vHuman measures, even though vHPCs are identical in both systems.

The dependency of mean vC2 removal rates on location within vLivers (Fig 7G–7I) is a consequence of upstream removal of vC2 (Fig 7H), the fact that the number of vHPCs decreases PV-to-CV, and the intra-Layer edges connecting SSs within Layers 0–3. The latter causes the length of the PV-to-PC path taken by some vCompounds to be much longer than the shortest, most direct PV-to-PC path. The magnitude of the differences within each of the three bands increases with decreasing *pEnter*. To illustrate, for *pEnter* = 1.0 (0.05), the PC/PP ratio for peak Entry rates (Fig 7G) is 1.4 (3.5). For percent of Dose per vHPC at *t* = 7,500 TS (Fig 7I) when using *pEnter* = 1.0 (0.05), the PC/PP ratio is 1.5 (2.8). The consequences of changing *pEnter* on vHPC Entry rates and cumulative removal are clearly evident within the PP band (Fig 7G and 7H). The influence of decreasing *pEnter* diminishes within the CL band and is almost absent within the PC band. Although the amounts of vC2 removed within the three bands decrease PP-to-PC, the per vHPC removal rates (Fig 7G), and thus per vHPC amounts (Fig 7I), increase PP-to-PC, which means that the PC vHPCs do more of the vC2 removal work, unlike in vCultures. Those observations may aid in disentangling explanations of IVIVE underpredictions.

In vCulture and vHuman experiments, a step-like pattern is evident in post-peak measures of removal rate (Figs 6B and 7B). That pattern is a consequence in part of specifying that the mean SS length in vHuman experiments be approximately 5 grid spaces, which is the same as the length of all Hepatocyte Spaces used by vCulture. Making those lengths equivalent results in both systems containing similar numbers of vHPCs, facilitating direct comparisons of results from vCulture and vHuman experiments. When each SS’s length is MC-sampled from a wider distribution, that step-like pattern vanishes [14].

### vCompound-3 achieves the cross-system validation target

**Fig 8:**
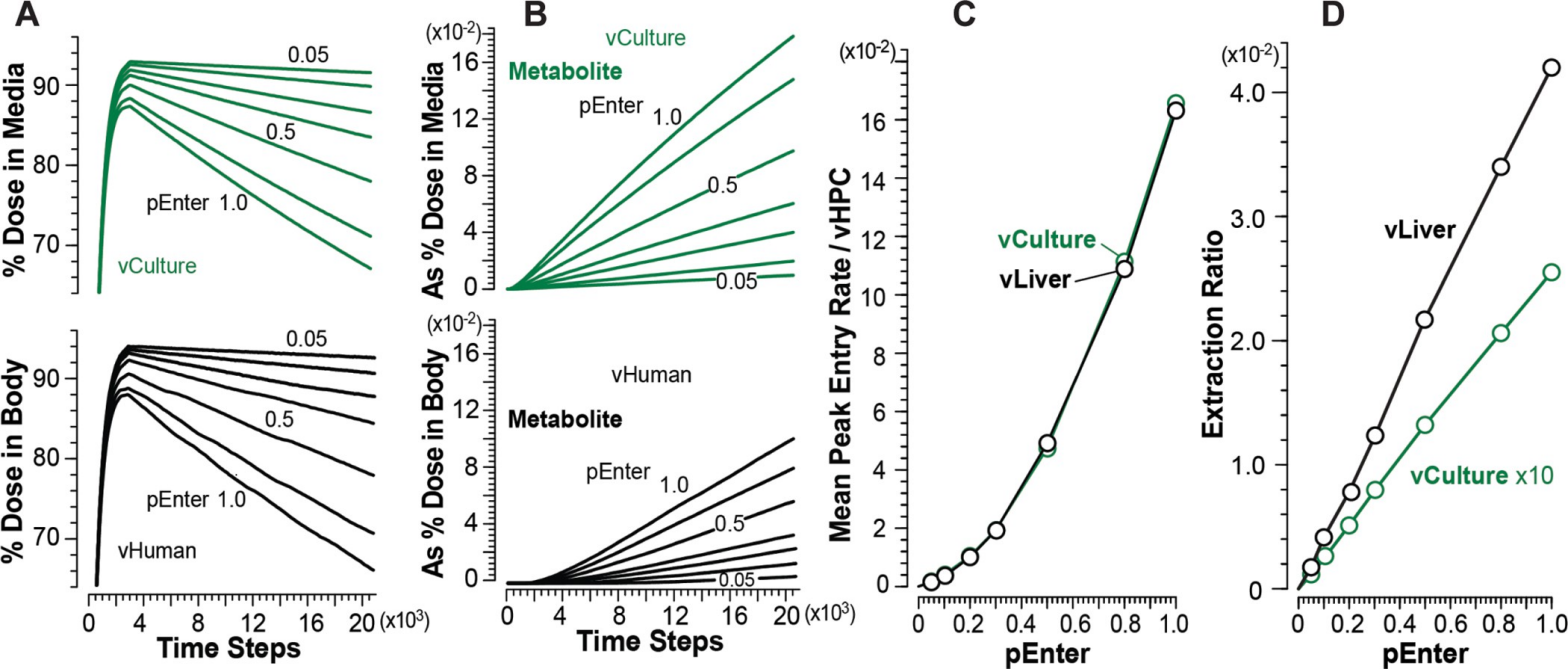
Results from vCulture and vHuman experiments using vC3. (A) Temporal measures of percent Dose in Body (top) and Media (bottom) for each *pEnter*. (B) Amount of Metabolite in Body (top) and Media (bottom) as percent of Dose for each *pEnter*. (C) Correlations between mean peak Entry rates and *pEnter*. (D) Correlations between mean Extraction Ratios and *pEnter*.

Measures of vC3 disposition and Metabolism during vCulture and vHuman experiments are provided in Fig 8. vC3 is intended to represent a highly permeable but very slowly metabolized xenobiotic. It uses *pBind* = 0.01 and *pMetabolize* = 0.005. Otherwise, the parameters that determine vC3 dynamics in both systems are identical to those used by vC1. For all *pEnter*, the percent of Dose in Media and Body (Fig 8A) are equivalent within the variance of 12 MC executions. Metabolite accumulates faster in Media than in Body (Fig 8B) because Metabolite in vLiver must traverse additional spaces and downstream SSs before exiting to CV and moving to Body.

We can infer from vC1 results that, because vC3’s rate of Metabolism is small, Entry rates will be approximately equivalent in both systems, which is the case (Fig 8C). The explanation for the nonlinearity in Fig 8C is the same as that provided above for vC1. Thus, the cross-system validation target is achieved for all *pEnter*, and vCulture measures directly predict corresponding vHuman measures.

During vHuman experiments, mean peak Entry rates increase PV-to-CV similar to increases measured for vC1 (Fig 5F). To illustrate for *pEnter* = 1.0 (0.1), the PC/PP ratio for mean peak Entry rate is 3.6 (4.4). The expected large between-system difference in mean Extraction Ratios (Fig 8D) is a combined consequence of three vLiver features: 1) the upstream Binding of vC3 to Enzymes; 2) the number of vHPCs decreases PV-to-CV, which increases downstream per vHPC exposures, as demonstrated in Fig 5F–5H; and 3) the intra-Layer edges connecting SSs within Layers 0–3. The latter causes the PV-to-PC path taken by some vCompounds to be much longer than the shortest PV-to-PC path illustrated in Fig 3B.

### vCompound-4 does not achieve the cross-system validation target

**Fig 9:**
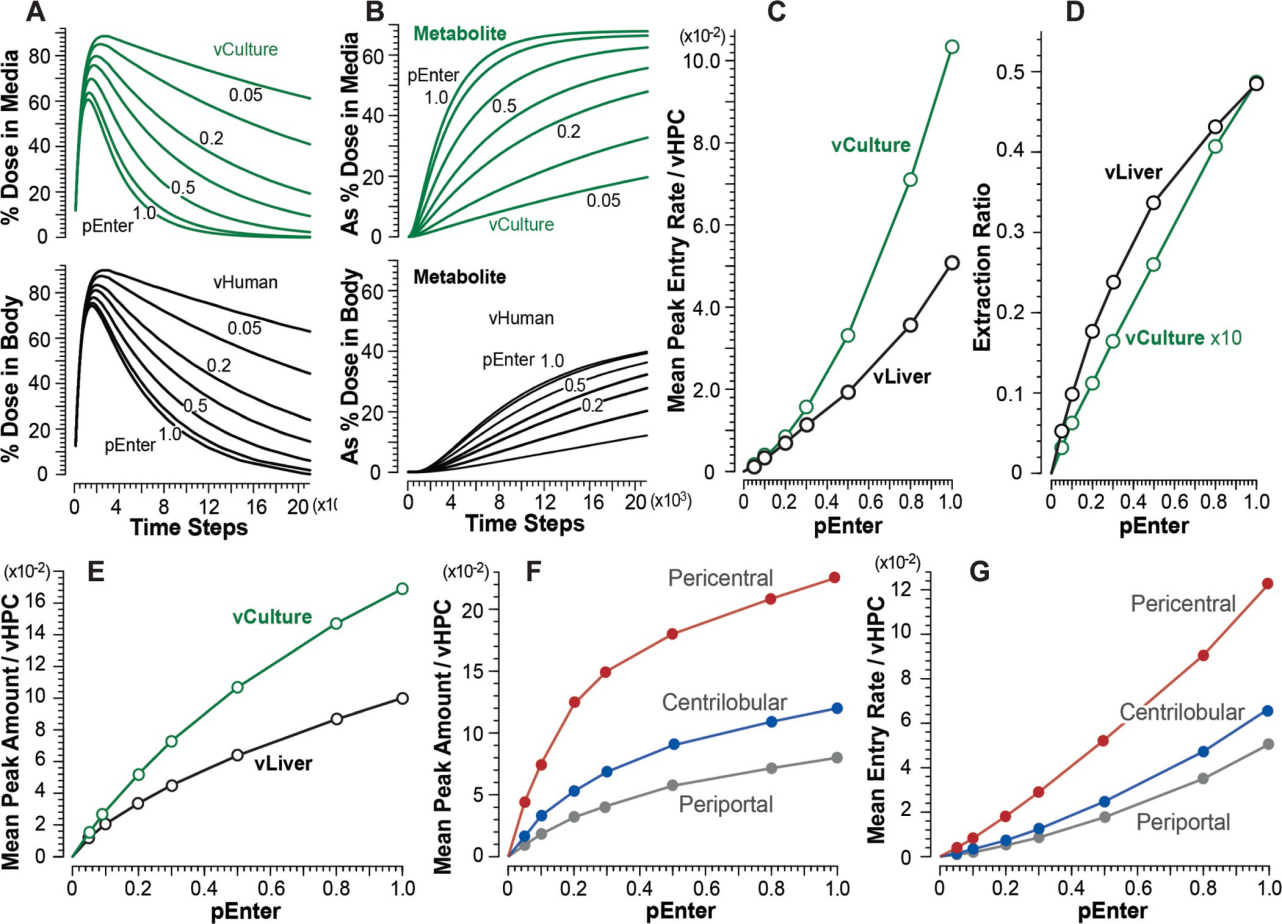
Results from vCulture and vHuman experiments using vC4. (A) Temporal measures of percent Dose in Body (top) and Media (bottom) for each *pEnter*. (B) Amount of Metabolite in Body (top) and Media (bottom) as percent of Dose for each *pEnter*. (C) Correlations between mean peak Entry rates and *pEnter*. (D) Correlations between mean plateau values of Extraction Ratio and *pEnter*. (E) Correlations between systemwide mean peak amounts of vC4 (objects) per vHPCs and *pEnter*. (F) Correlations between mean peak amounts of vC4 per vHPCs within the three vLiver bands and *pEnter*. (G) Correlations between mean peak Entry rates within the three bands and *pEnter*.

vC4 represents a highly permeable xenobiotic having an intermediate hepatic extraction ratio. It uses *pBind* = 0.1 and *pMetabolize* = 0.015. The extra-vHPC behaviors of vC3 and vC4 Metabolites are identical. The results in Fig 9A show that, for all values of *pEnter*, the rate of clearance of vC4 from Media is larger than from Body. The magnitude of the difference increases with increasing *pEnter*. As for vC3, Metabolite accumulates faster in Media than in Body (Fig 9B) because Metabolite in vLiver must traverse additional spaces and downstream SSs before exiting to CV and moving to Body.

The results in Fig 9C show that mean peak Entry rates for vCulture overpredict corresponding vHuman values, and the magnitude of the overprediction decreases with decreasing values of *pEnter*. As examples, for *pEnter* = 1.0 (0.1), mean peak Entry rates for vCulture overpredict corresponding vHuman values by 2.0-fold (1.2-fold). The explanation for those overpredictions highlights systemic differences in the temporal mixing of disposition and intra-vHPC events between the two systems. Each TS during early intervals, a fraction of the Dose in Media Space is transferred randomly to 114 Media-Cell Interfaces, whereas, for Body, the fraction is transferred randomly to only 45 SS. During vCulture experiments, a vC4 may enter and exit only two spaces, Media-Cell Interface Space and Hepatocyte Space, whereas in vLivers, between entering and exiting an SS, a vC4 may enter and exit additional spaces: Core, Interface, Endothelial Cell Space, and Space of Disse. Also, during the average interval that a vC4 requires to enter and exit a vLiver SS, a vC4 may cycle two or more times between Media and Media-Cell Interface Space.

vHPC Entry and Enzyme binding events are correlated. Results in Fig 9E show that, for vCulture, system-wide mean peak amounts of vC4 per vHPC are larger than corresponding system-wide mean peak amounts in vLiver. Thus, system-wide, at corresponding times, more vC4 Metabolism has occurred in vCulture experiments. During comparable intervals, Metabolic events per vHPC within vCulture and within vLiver’s PP band are similar. However, within vLiver, Metabolic events per vHPC increase PP-to-CL-to-CV (Fig 9F and 9G).

In both systems, mean values of Extraction Ratio plateau by about 7,500 TS. The large difference in Extraction Ratios is a consequence of vLiver-vCulture structural differences. In vCulture, a vC4 that is bound to an Enzyme and released later without being Metabolized is, on average, unlikely to become Enzyme-bound and Metabolized within another vHPC before returning to Media. However, in vLiver, a vC4 that is bound to an Enzyme in an upstream vHPC and later released unchanged will, on average, have additional opportunities to be Metabolized before returning to Body, as evidenced by peak amounts per vHPC (Fig 9F) and Entry rates (Fig 9G) increasing from PP to PC bands.

On average, during a vCulture experiment using *pEnter* = 1.0, the mean peak Entry rate is 0.102, whereas during a vHuman experiment, the corresponding values within the PP, CL, and PC bands are 0.051, 0.066, and 0.12, respectively. That PP-to-PC increase is larger for smaller *pEnter* values, similar to increases measured for vC1 (Fig 5F). To illustrate for *pEnter* = 1.0 (0.1), mean peak Entry rates increase 2.35-fold (3.6-fold) from the PP to the PC band. The patterns for the correlation between mean peak Entry rates within the PP, CL, and PC bands (Fig 9G) are similar to corresponding patterns for vC1 (Fig 5F), although Entry rates of the former are smaller due to upstream binding of vC4s to Enzymes and cumulative Metabolism.

## Discussion

The work presented marks the first step in demonstrating the feasibility of the plan illustrated in Fig 1. To provide a solid foundation for exploiting the plan’s full potential, it is essential to demonstrate that the cross-system validation target is achievable: when using matched dosing conditions, a 1:1 equivalency exists for specific vCompound types between measures of unbound vCompound Entry rates (per vHPC) made during vCulture and vHuman experiments. In those cases, vCulture Entry rates directly predict the corresponding measures made during vHuman experiments. From that set of idealized conditions, one can in parallel incrementally refine vCulture and vHuman parameterizations to achieve Stage One and Two validation targets. Differences in the resulting vCulture and vHuman model mechanisms may provide a plausible multi-feature accounting for the discrepant IVIVE prediction of hepatic clearance. In some cases, there may be multiple equally plausible yet different (in one or more feature parameterizations) explanations. When needed, wet-lab experiments can be used to challenge and possibly falsify one or more.

For the four vCompound types studied, we achieved the 1:1 equivalency in 15 of 28 cases: 1) the seven vC1 cases (*pEnter* = 0.05–1.0) where the vCompound removal rate is zero; 2) the seven vC3 cases where vCompound removal rates are much smaller than vHPC Entry rates (maps to a slowly metabolized xenobiotic); and 3) in one vC2 case where *pEnter* = 1.0 and thus removal rates ≅ Entry rates. For the other 13 cases, differences in the structural organization of vHPCs within the vCulture and vHuman systems cause vCulture measures to either underpredict (vC2, *pEnter* ≤ 0.8) or overpredict (vC4, all cases) corresponding vHuman measures. The magnitude of the vC2 underpredictions increase as *pEnter* decreases (Fig 7C). It is noteworthy that when using *pEnter* = 0.05, even though vCulture-vHuman operational differences are minimized, mean peak Entry rates (and areas under percent Dose in Media curves) in vCulture experiments underpredict corresponding vHuman measures by a factor of 4.4. The differences between the cumulative removal profiles (Figs 6F and 7F) clearly show that, for small *pEnter* values, the structural organization of the vLiver is strikingly more efficient at removing vC2.

We recorded and measured temporal differences in event details as they unfolded at different locations within each system. Using that information, we developed explanations for how and why vCulture measures either under- or overpredicted corresponding vHuman measures. Those explanations demonstrate how study of concretized model mechanisms can facilitate deep thinking about the actual mechanisms responsible for IVIVE discrepancies.

Previous reports detail strengths and weaknesses of the analogical approach and methods employed herein [7, 9, 10, 14, 17, 28]. We argue that, because of measurement limitations and the fog of multi-source uncertainties impacting IVIVEs, increased reliance on analogical arguments may be necessary to make progress disentangling mechanisms contributing to IVIVE inaccuracies. Bartha provides guidelines for assessing the scientific acceptability and limitations of analogical arguments [18]. Reliance on analogical arguments and reasoning can be both a limitation and strength [19].

Most IVIVE methods employ either the well-stirred (most common) or parallel tube liver model even though researchers understand that neither model can represent liver physiology complexities, such as the heterogeneity in enzymatic expression (zonation) [29, 30]. Those liver models continue to be used because doing so limits the mathematical complexities of the methods employed. Given the results presented, the following conjecture is reasonable. Abstracting away critical structural information about hepatocyte organization within the liver contributes to discrepant IVIVE predictions of hepatic clearance in humans.

The highly permeable vC1-vC4 are a tiny sample from the spaces of vCompound types and their xenobiotic referents. Additional work will be needed to explore vCulture-vHuman differences in disposition, removal, and Entry rate measures for a diverse variety of vCompound types. Disposition-influencing parameters, which are specific to a vCompound type, are *pEnter* and *pExit* (each value can also be Cell-type-specific), and *forward-* and *lateralBias* (which can vary within different SS Spaces). Within vHPCs, the Enzyme type, *bindersPerCell(Min/Max)*, and parameters, *pBind*, *bindCycles*, *pMetabolize*, and *bindersPerCell(Min/Max)* can be vCompound-type-specific. Transport parameters can be included as part of future explorations and made vCompound-type-specific without requiring vLiver structural changes. vCompound types utilizing different values and combinations of those parameters may amplify or diminish the over- and underpredictions described above.

There is ample evidence that in vitro-in vivo differences in hepatocyte heterogeneity contribute significantly to IVIVE prediction discrepancies [3, 4, 30, 31], yet the vCulture and vHuman experiments employed only homogeneous vHPCs. In doing so, we provide the necessary and essential foundation for future research that is needed to explore vCulture-vHuman differences caused by vHPC heterogeneity. Expression levels increase PP-to-PC for most liver genes responsible for detoxification and xenobiotic metabolism [32]. We anticipate that exploration of PP-to-PC difference (zonation) in vHPC-vCompound interaction consequences will be required to meet many xenobiotic-specific Stage Two validation targets. The vLiver’s design enables simulating such location-dependent model mechanisms [12, 23].

Because of the structural organization of vHPCs within vLivers, vHPC Entry rates increase PP-to-PC for all four vCompounds, notwithstanding that all vHPCs are identical, and, in the case of vC2, where each Entry event is also a removal event. Additional work is needed to explore whether the degree of interaction between Metabolism and increasing PP-to-PC Entry rates amplifies discrepant IVIVE predictions of hepatic clearance.

We anticipate that achieving Stage Two validation targets for many xenobiotics will require that Metabolism increase PP-to-PC. The resulting increases in model mechanism complexity will require clear evidence that code changes have not compromised cross-system validation requirements. Code changes are often necessary during iterative refinement (e.g., see [25]). Inclusion of a second vCompound, e.g., one of those studied here, can provide that evidence. To illustrate, we note in Methods that each Dose includes equal amounts of Marker (a multi-attribute internal standard) and vCompound. If we repeat vCulture and vHuman experiments using Dose = vC2 + vC1 rather than Dose = vC2 + Marker, measures of vC2 dynamics in both experiments will be the same (within experiment variance). Further, vC1 measures will be the same as those in an experiment in which Dose = vC1 + Marker. To facilitate achieving the Fig 1 plan, we can replace Marker with a vCompound from the vC1-vC4 set. To illustrate, consider a set of Stage Two experiments. Dose contains a 50:50 mix of a new vCompound and vC1 (using *pEnter* = 1) in place of Marker. Parameterizations of the new vCompound will be refined iteratively to achieve Stage One and Two validation targets for the referent xenobiotic. During that process, vC1 properties are expected to be invariant. Upon concluding each experiment, vC1 measurements, such as those in Figs 4 and 5, are plotted along with new vCompound measurements. Any significant change in vC1 measurements from one refinement cycle to the next serves as a red flag. In such a case, the software issue is corrected, the necessary system verification tests are concluded, and iterative refinement continues. Because Metabolism will be a focus for all Stage Two experiments, we anticipate that it will be more informative to use a Metabolized vCompound, such as vC4, as the internal standard, rather than vC1. During parameter refinement, the PP-to-PC pattern of Metabolism of a new vCompound can be adjusted, while keeping that pattern invariant for vC4.

Once Stage One and Two validation targets have been achieved for several xenobiotics, quantitative mappings can be established between a subset of xenobiotic physicochemical properties (or molecular descriptors calculated from structure information) and the set of vCompound-specific parameter values listed above. Using an earlier version of the vLiver, Yan et al. identified different sets of vCompound-specific parameter values that enabled quantitative validation against liver perfusion data for four drugs [5]. The authors then used those relationships to successfully predict vCompound-specific parameter values for two additional drugs, given only values of each drug’s physicochemical properties. The authors discuss the strengths, weaknesses, and limitations of using those particular methods to predict vCompound-specific parameter values, given only values of a new xenobiotic’s physicochemical properties.

In summary, the results support the feasibility of using vCulture and vHuman model mechanisms to limit observed IVIVE discrepancies. That support is a consequence of three vLiver and vCulture characteristics. 1) The structural organizations of vHPCs within the two systems are strongly analogous to that in their referents. 2) Model mechanisms, including vCompound removal by vHPCs can be parameterized to be biomimetic. 3) Mean measures of vCompound dynamics can be scaled to match corresponding wet-lab measurements within prespecified criteria. Supportive, use-case-specific evidence is provided in five reports [8, 10–12, 14]. Further evaluations are needed near term to characterize within- and cross-system consequences of reducing Cell permeability and making vHPCs heterogenous.

## Supporting information

S1 Table Key vCompound parameter values and specifications for Monte Carlo experiments and structural features. Table subsections for vHuman and vCulture: Experiment; vCompound features, events, & activities; vCompound convection and dispersion; and Structural features.
(PDF)
