# Supplementary material for: Utilizing virtual experiments to increase understanding of discrepancies involving in vitro-to-in vivo predictions of hepatic clearance
Source: PLoS One. 2022 Jul 22;17(7):e0269775. doi: 10.1371/journal.pone.0269775 (PMC9307204; doi:10.1371/journal.pone.0269775)
Supplement: S1 Table — Table subsections for vHuman and vCulture: Experiment; vCompound features, events, & activities; vCompound convection and dispersion; and Structural features. (PDF) [file pone.0269775.s001.pdf]

Utilizing virtual experiments to increase understanding of discrepancies involving in vitro-in vivo predictions of hepatic clearance

Supporting S1 Table

Key vCompound parameter values and specifications for MC experiments and structural features.

Experiment	vHuman	vCulture
Number of MC executions	12	12
Number of time steps	21600	21600

vCompound<br>Features, Events, & Activities	vHuman	vCulture
Marker (Membrane crossing)	FALSE	FALSE
vC1 (Membrane crossing)	TRUE	TRUE
pEnter	[0.05, 0.1, 0.2, 0.3, 0.5, 0.8, 1.0]	[0.05, 0.1, 0.2, 0.3, 0.5, 0.8, 1.0]
pExit	1	1
vC2 (Membrane crossing)	TRUE	TRUE
pEnter	[0.05, 0.1, 0.2, 0.3, 0.5, 0.8, 1.0]	[0.05, 0.1, 0.2, 0.3, 0.5, 0.8, 1.0]
pExit	1	1
vC3 (Membrane crossing)	TRUE	TRUE
pEnter	[0.05, 0.1, 0.2, 0.3, 0.5, 0.8, 1.0]	[0.05, 0.1, 0.2, 0.3, 0.5, 0.8, 1.0]
pExit	1	1
vC4 (Membrane crossing)	TRUE	TRUE
pEnter	[0.05, 0.1, 0.2, 0.3, 0.5, 0.8, 1.0]	[0.05, 0.1, 0.2, 0.3, 0.5, 0.8, 1.0]
pExit	1	1

vCompound<br>Convection, Dispersion	vHuman	vCulture
innerForwardBias	0.2	0.2
innerLateralBias	0.5	0.5
outerForwardBias	0.2	0.2
outerLateralBias	0.5	0.5
ssFlowRate	1	1

Utilizing virtual experiments to increase understanding of discrepancies involving in vitro-in vivo predictions of hepatic clearance

S1 Table (cont.)

Structural Features	vHuman	vCulture
	vLiver, Body, and Intro space (to contain Dose)	Not used: Core, Endothelial Cell Space, and Space of Disse
Edges	55 Layer 0-to-1; 65 L1-to-L2; 35 L2-to-L3; 25 L3-to-L4 edges.	Media-Cell Interface = merged (Interface Space, PV, CV)
	Intra-Layer edges:	
	Layer 0 =20, Layer 1=7, Layer 2=5, Layer 3=2, Layer 4=0	
Width (grid spaces)	SS = 15	Hepatocyte Space = 15
Length (grid spaces)	SS = 5 ± 1(SD)	Hepatocyte Space = 5
MC Sampled per experiment	12 vLobules	114 Hepatocyte Spaces
vHPC's per execution	Mean = 8,475 (SD = 167)	8,550
Dose	Single	Single
Marker	50,000	50,000
vCompound	50,000	50,000
SVN Version ID	1352	1352
